# Childhood hyperactivity, eating behaviours, and executive functions: Their association with the development of eating-disorder symptoms in adolescence

**DOI:** 10.1186/s40337-023-00902-z

**Published:** 2023-10-13

**Authors:** Rachel Dufour, Édith Breton, Alexandre J. S. Morin, Sylvana M. Côté, Lise Dubois, Frank Vitaro, Michel Boivin, Richard E. Tremblay, Linda Booij

**Affiliations:** 1 Sainte-Justine Hospital Research Centre, Montreal, Canada; 2 Department of Psychology, Concordia University, Montreal, Canada; 3 Department of Psychiatry and Addictology, Université de Montréal, Montreal, Canada; 4 School of Public Health, Université de Montréal, Montreal, Canada; 5 School of Epidemiology and Public Health, University of Ottawa, Ottawa, Canada; 6 School of Psychoeducation, Université de Montréal, Montreal, Canada; 7 Department of Psychology, Université Laval, Québec City, QC Canada; 8 Department of Psychology and Pediatrics, Université de Montréal, Montreal, Canada; 9 Department of Psychiatry, McGill University, Montreal, Canada; 10 Research centre, Douglas Mental Health University Institute, Montreal, Canada; 11 Eating Disorders Continuum, Douglas Mental Health University Institute, Montreal West Island Integrated University Health and Social Service Centre, 6603-05 LaSalle Blvd, Montreal, QC H4H 1R3 Canada

**Keywords:** Eating disorders, Hyperactivity, Executive functions, Adolescence, Childhood eating

## Abstract

**Background:**

Cross-sectional studies have shown that hyperactivity and impaired executive functioning are associated with symptoms of eating disorders in adolescence and adulthood. Whether hyperactivity and executive functions in early life can prospectively predict the emergence of eating disorder symptoms in adolescence remains unknown. The present study relies on a longitudinal design to investigate how hyperactivity at age 3, eating behaviours at age 3.5 and cognition at ages 3–6 were associated with the development of eating-disorder symptoms from 12 to 20 years old.

**Methods:**

Using archival data collected since 1997 from the Quebec Longitudinal Study of Child Development cohort (*N* = 2, 223), we used Latent Curve Models to analyse predictors of youth’s trajectories of eating-disorder symptoms at four timepoints.

**Results:**

A quadratic (curvilinear) trajectory of eating-disorder symptoms was found to be most representative of the data. Higher hyperactivity at age 3 was associated with higher levels of eating-disorder symptoms at age 12, and this association was partially mediated by higher levels of overeating and cognitive inflexibility in childhood. Cognitive inflexibility in childhood also mediated the association between hyperactivity at age 3 and increases in eating-disorder symptoms during adolescence. Furthermore, working memory was indirectly related to eating-disorder symptoms via the mediational role of cognitive flexibility.

**Conclusions:**

Hyperactivity, overeating, cognitive inflexibility, and working memory early in life might precede the onset of eating-disorder symptoms in adolescence. Early behavioural and cognitive screening may help to identify children who are most at risk for eating disorders. This, in turn, could guide preventive interventions.

**Supplementary Information:**

The online version contains supplementary material available at 10.1186/s40337-023-00902-z.

## Abstract (plain language summary)

Eating-disorder symptoms, such as body image issues, maladaptive behaviors, and preoccupation with weight, tend to develop in adolescence. However, it is unclear whether early childhood characteristics or behaviours could be indicators of a risk of developing eating-disorder symptoms later. The current study examined the possible link between certain early behaviours (e.g., hyperactivity, childhood eating), early cognitive processes, and eating-disorder symptoms development in a community cohort followed from birth. Results showed that being hyperactive in early childhood predicts higher levels of eating-disorder symptoms at the beginning of adolescence (age 15), and that this is partially explained by a link between being hyperactive, being more rigid in our ways of thinking, and engaging in overeating behaviours. Additionally, more early rigid ways of thinking predicted the increase in symptoms over time. Our results demonstrate possible behaviours and characteristics that could be used to identify children at risk of eating disorders, which in future research could potentially help improve our preventive interventions.

**Supplementary Information:**

The online version contains supplementary material available at 10.1186/s40337-023-00902-z.

## Introduction

Eating disorders (EDs) are debilitating and potentially life-threatening conditions associated with one of the highest treatment costs and mortality rates out of all psychiatric disorders [1–3]. Key ED symptoms include intense preoccupations with eating and weight, body image concerns, and maladaptive compensatory behaviours (e.g., self-induced vomiting) [4]. During adolescence, prevalence estimates of EDs range from 1 to 15%, whereas at least 30% of adolescent girls and 15% of adolescent boys display subthreshold symptoms of EDs [5–7]. A long history of subthreshold disordered eating may lead to the emergence of EDs that are resistant to treatment [8]. Therefore, identification of early risk factors and developmental processes underlying ED symptoms prior to the initial symptom presentation is important.

It is generally believed that early childhood environment and behaviours interact with a child’s neurodevelopment, thereby increasing the risk for the emergence of psychiatric conditions in early adulthood [9, 10]. Among the various possible early behaviours that have been associated with disordered eating, *childhood eating behaviours* and *hyperactivity* are considered highly relevant [4, 11, 12]. Their associations with the development of ED symptoms and their interactions in doing so remains understudied and unclear. Specifically, childhood overeating has been shown to predict binging-purging symptoms, whereas picky eating has been linked to the ED Anorexia Nervosa (AN) [13]. Likewise, researchers have also identified links between early attention deficit hyperactivity disorder (ADHD) symptoms, particularly the behavioural component of hyperactivity, and the emergence of EDs in adolescent and adult samples [14–18]. As a neurodevelopmental disorder, symptoms used to diagnose ADHD tend to be recognized in early childhood and persist across the lifespan [4]. Importantly, hyperactivity has been linked to overeating, although it is unclear if it also predicts picky eating [19]. However, the prospective association between hyperactivity in early childhood and the risk for EDs later in life remains unknown. As hyperactivity has mainly been associated with binging-purging disorders, and associations with restrictive EDs remain unclear, the ADHD component could represent a risk factor transdiagnostically.

In addition to examining behavioural predictors of ED symptoms, higher-order cognitive processes, also known as *executive functions*, could represent another type of early risk factors or early signs for EDs and be representative of alterations in neurodevelopment. Two central components of executive functioning are considered of interest due to their interactions with hyperactivity and EDs: working memory and cognitive flexibility. There is some evidence that people with Bulimia Nervosa (BN) have alterations in working memory, defined as the ability to hold and manipulate information in one’s mind [20]. Impaired cognitive flexibility (i.e., the ability to adapt and change one’s approach to problem solving) has also been reported among a portion of individuals with AN and eating disorder not otherwise specified (EDNOS), and appears to be independent of duration of illness or severity [20–22]. Findings are mixed regarding the presence of impaired cognitive flexibility in adolescent EDs and among other diagnostic categories such as BN [20, 23]. Additionally, ADHD symptoms have been linked to cognitive inflexibility and impaired working memory [24–26]. However, most studies conducted in this area are cross-sectional, making it impossible to clarify the direction of these associations. It also remains unclear how executive functions (particularly cognitive flexibility) and childhood eating behaviours such as overeating and picky eating could account for the links between hyperactivity and later EDs.

## Objectives and hypotheses

Using longitudinal latent curve modeling, we investigated the predictive role of hyperactivity, eating behaviours, and executive functions in childhood on trajectories of ED symptoms during adolescence. Our overall hypothesis was that hyperactivity, executive functions, and eating behaviours in childhood would be associated with different components of ED symptoms trajectories (i.e., initial level at 12, rise over time from 12 to 20, and shape of increase). It was hypothesized that **(1)** There will be considerable inter-individual variability in ED symptoms trajectories during adolescence and that (**2)** greater hyperactivity, poorer executive functions (i.e., working memory and cognitive flexibility), and greater childhood eating behaviours (i.e., overeating and picky eating) would predict higher initial levels and growth over time in ED symptoms trajectories during adolescence/early adulthood. Furthermore, (**3)** we analyzed whether childhood eating behaviours and cognitive flexibility, a core component of executive functioning known to be prevalent in individuals with AN, would mediate the association between hyperactivity at age 3.5 and ED symptoms in adolescence. Identifying early risk factors for ED symptom development could be useful for the development of early prevention programs for EDs.

## Methods

### Participants and design

This study relies on archival data from the Quebec Longitudinal Study of Child Development (QLSCD) cohort. In 1997–1998, 2223 participants were recruited randomly at the age of 5 months through the Quebec Master Birth registry [27, 28]. Participants have since been followed every one to two years. For this study, the time points of interest were collected when participants were aged 41 months, 44−56 months, 6 years, 12 years, 15 years, 17 years, and 20 years. This study was approved by the Health Research Ethics Committee of the Quebec Statistics Institute, the Research Ethics Board of the Sainte-Justine University Hospital Center, and the Concordia University Research Ethics Committee. 1996 participants completed at least one of the measures (48.8% girls, 51.2% boys). The ethnicity of these participants was distributed as follows: Canadian (*n* = 1 447, 72.5%), French (*n* = 653, 32.7%), British (*n* = 144, 7.2%), European (*n* = 176, 8.8%), Indigenous (*n* = 56, 2.8%), African or Haitian (*n* = 43, 2.2%), and other (*n* = 265, 13.3%). The amount of missing data was 12.5 to 15.8% for the early childhood measures (missing at random) but was higher for variables collected in adolescence/early adulthood (35.1 to 44.1%), which is expected considering the longitudinal study design. Boys were slightly more likely to have missing data in adolescence than girls, thus missing data were not missing at random for the adolescence variables. Individuals with missing all data in adolescence did not differ in terms of data availability for the childhood predictors.

## Measures

### Hyperactivity

Hyperactivity was measured at 41 months using the five relevant items from the Interviewer Computerized Questionnaire, which is composed of elements from the Child Behavior Checklist, the Ontario Child Health Study Scales, and the Preschool Behavior Questionnaire [29]. These items were: (1) Cannot stay in place, is agitated? (2) Stirs constantly? (3) Has been impulsive, acting without thinking? (4) Difficulty waiting its turn in a game? (5) Has difficulty staying calm? Mothers or primary caregivers reported whether the item applied to their child by selecting either “*Never or not true”* (1), “*Sometimes true”* (2) or “*Often to very true”* (3). Original questionnaires where these items were taken from have been shown to have good reliability (*a* = 0.87) and test–retest reliability (*r* = 0.76) [30]. In our sample, this scale had moderate scale score reliability (Cronbach’s alpha = 0.72).

### Working memory

The imitation sorting task was used to assess working memory [31] at 41 months. In this game of imitation, the child is asked to reproduce different arrangements that are showed progressively and sorted into two containers. Every child completed four levels of the task. Each level was scored as either “*Success”* (1) or “*Failure”* (0) by the examiner. For this study, we used the total number of successes as an observed measure of working memory level. Psychometric properties of this measure are adequate [31] and this task has been developed for assessing working memory in very young children, although scale score reliability in our sample was quite poor (Cronbach’s alpha = 0.50).

### Cognitive flexibility

The figural intersection task was used to assess cognitive flexibility [32] at 6 years. Every child completed 8 levels of the task. During this task, the child is asked to identify the intersection of relevant shapes when they appear overlapping. The size and orientation of the shapes change, and the child is exposed to irrelevant shapes that they must ignore when presented with new relevant shapes. Each level was scored as either “*Success”* (1) or “*Failure”* (0) by the examiner. For this study, we used the total number of successes as an observed measure of their cognitive flexibility level. This task has been shown to be a reliable measure of mental capacity, inhibition, flexibility, and speed processing [32, 33]. Psychometric research suggests that scores on this test have adequate scale score reliability (Cronbach’s alpha = 0.79) and construct validity [32, 33].

### Childhood eating behaviours

Overeating and picky eating were assessed during preschool when children where aged between 44 and 56 months, based on maternal report (see www.iamillbe.stat.gouv.qc.ca for more information). An expert committee on nutrition, including researchers and practitioners, reviewed the eating behaviours questionnaire, which was also pre-tested in an independent sample of parents with preschool-age children [34, 35]. **Overeating** was measured using two items: [1] Does your child eat too fast, and (2) Does your child eat too much (correlation between the two items; *r* = 0.45). **Picky eating** was measured using two items: (1) Is your child difficult with food, and (2) Does your child refuse to eat (*r* = 0.52). Mothers rated all items as either *“never (1)” “rarely (2)” sometimes (3)”* or* “often (4)”.*

### Eating disorder symptoms

The Sick, Control, One stone, Fat, Food (SCOFF) questionnaire was administered to assess ED symptoms at 12, 15, 17, and 20 years old [36, 37]. and includes the following items: (1) Do you make yourself sick because you feel uncomfortably full? (i.e., purging) (2) Do you worry that you have lost control over how much you eat? (i.e., loss-of-control eating) (3) Have you recently lost more than 6 kg in a 3-month period? (i.e., weight loss) (4) Do you believe yourself to be fat when others say you are too thin? (i.e., feeling overweight) (5) Would you say that food dominates your life? (i.e., attributing importance to food). Responses to these items were coded as “yes (1)” or “no (0)”. At a cut-off of two, sensitivity (94.6%) and specificity (94.7%) have been shown to be excellent [37]. Using the items non-dichotomously, Cronbach alphas at four timepoints in our sample averaging 0.74 demonstrate adequate scale score reliability.

## Statistical analyses

Analyses were done in *Mplus* 8.8 [38] using robust diagonally weighted least square estimation (WLSMV) to account for the ordinal nature of the indicators (which all include less than 5 response categories and some binary indicators; [39]), and the theta parameterization. All models were estimated based on the full information available, relying on algorithm implemented in *Mplus* for WLSMV estimation to handle missing data using Pairwise Present [40], allowing us to capitalize on the whole sample [41]. Preliminary measurement models for each construct and longitudinal measurement invariance of ED symptoms were examined (Additional file 1).

### Latent curve modeling (LCM)

To model participants’ trajectories of ED symptoms over the course of adolescence, we used LCM. In these models, we set the scale of the factors by fixing the loading of a referent indicator to 1 in order to retain the natural scaling of the measure. However, a similar approach could not be retained for the mean structure. As a result, we set the mean scale of the factors by freely estimating all thresholds (while maintaining strong invariance, and thus constraining them to equality over time) and fixing the mean of the Time 1 (age 12) factor, and thus of the LCM intercept factor, to 0. As a result, our trajectories can be interpreted as reflecting the natural scaling of our measure but centered around a grand mean of 0 at Time 1 (age 12). We first estimated a linear LCM, with time codes reflecting the passage of time in yearly intervals (0, 3, 5, 8) for an intercept located at 12 years. We contrasted this model with a quadratic LCM, in which a quadratic slope factor was added and defined based on squared timecodes (0, 9, 25, 64). These two models were compared based on model fit and parameter estimates to locate the optimal representation of ED trajectories.

### Predictive analyses and mediation

To test the associations between our predictors and the ED growth factors, we included CFA factors representing hyperactivity, overeating, and picky eating as well as observed variables reflecting working memory and cognitive flexibility to the optimal LCM solution. We contrasted a solution of partial mediation to one of full mediation. In both models, working memory was allowed to predict the growth factors as well as the mediators (overeating, picky eating, and cognitive flexibility), as we had no hypothesis regarding mediation in relation to this distal predictor. In both models, hyperactivity was specified as a predictor of the three mediators. Direct links between hyperactivity and the growth factors were also added to the model of partial mediation. Lastly, the three mediators were allowed to correlate with one another and to predict the growth factors in both models. To test for mediation, we relied on the Mplus model INDIRECT function to test the statistical significance of the indirect effects of hyperactivity on the growth factors as mediated by overeating, picky eating, and cognitive flexibility. More specifically, the significance of these indirect effects was calculated using 95% bias-corrected bootstrapped confidence intervals (using 1000 bootstrap samples), which indicate statistical significance when they exclude 0.^1^

Given the documented sex differences in the prevalence of ED symptoms and hyperactivity [2, 4, 5, 42–44], supplementary analyses of measurement invariance and equivalence (e.g., [45, 46]) were considered and reported in Additional file 2.

## Results

Fit indices of our measurement models are outlined in Table 1. The global measurement model had an excellent fit to the data. The results further supported the equivalence of model form and of item intercepts and thresholds (i.e., configural and strong invariance) of the ED factors over time as well as the invariance of their variance, meaning ED factors measurement properties are equivalent across the four timepoints. The parameter estimates from our most invariant model are reported in Table 2 and reveal well-defined factors with satisfactory estimates of composite reliability, especially if we account for the reduced length of these scales and our reliance on fully latent models corrected for measurement errors [47]. They also support the distinctiveness of our constructs and highlight how the rank-order stability of ED symptoms seems to increase over time.

**Table 1:** Results from the Measurement Invariance Models

Model	*χ*^*2*^ (df)	CFI	TLI	RMSEA	90% CI	CM	ΔCFI	ΔTLI	ΔRMSEA	Δ*χ*^*2*^ (df)
*Measurement models*										
1. Total/configural	562.107 (368)*	0.973	0.966	0.019	0.016; 0.022	–	**–**	**–**	**–**	**–**
2. Strong	600.847 (337)*	0.969	0.963	0.020	0.017; 0.022	1	− 0.004	− 0.003	+ 0.001	37.401 (9)*
3. Strict	995.875 (352)*	0.925	0.914	0.030	0.028; 0.032	2	− 0.044	− 0.049	+ 0.010	382.381 (15)*
3a. Partial strict	683.375 (351)*	0.961	0.955	0.022	0.019; 0.024	2	− 0.008	− 0.008	+ 0.002	83.117 (14)*
4. Latent variance	718.804 (354)*	0.958	0.951	0.023	0.020; 0.025	3a	− 0.003	− 0.004	+ 0.001	27.705 (3)*
5. Latent means	1035.677 (357)*	0.921	0.910	0.031	0.029; 0.033	4	− 0.037	− 0.041	+ 0.008	325.984 (3)*
*Latent curve models*										
L1. Linear	542.205 (162)*	0.911	0.896	0.038	0.034; 0.041	–	**–**	**–**	**–**	**–**
L2. Quadratic	361.787 (158)*	0.953	0.943	0.028	0.024; 0.032	L1	+ 0.042	+ 0.047	− 0.010	136.390 (4)*
*Predictive latent curve models*										
P1. Full Med	779.885 (404)*	0.957	0.950	0.022	0.019; 0.024	–	**–**	**–**	**–**	**–**
P2. Partial Med	777.268 (401)*	0.957	0.950	0.022	0.019; 0.024	P1	0.000	0.000	0.000	6.314 (3)

^*^ *p* ≤ .01; *χ*^*2*^ = chi-square test of exact fit; df = degrees of freedom; CFI = comparative fit index; TLI = Tucker-Lewis index; RMSEA = root mean square error of approximation; 90% CI: 90% confidence interval for the RMSEA; CM = comparison model; Δ = change in model fit relative to the CM

**Table 2:** Standardized Factor Loadings, Uniquenesses, Correlations, and Composite Reliability

Item	Hyperactivity	OE	PE	ED at 12	ED at 15	ED at 17	ED at 20
*Factor loadings*							
Item 1	0.814	0.669	0.723	0.329^1^	0.687	0.687	0.687
Item 2	0.831	0.669	0.723	0.859	0.859	0.859	0.859
Item 3	0.460			0.370	0.370	0.370	0.370
Item 4	0.460			0.549	0.549	0.549	0.549
Item 5	0.724			0.575	0.575	0.575	0.575
*Uniqueness*							
Item 1	0.337	0.552	0.478	0.892	0.528	0.528	0.528
Item 2	0.309	0.552	0.478	0.263	0.263	0.263	0.263
Item 3	0.788			0.863	0.863	0.863	0.863
Item 4	0.788			0.698	0.698	0.698	0.698
Item 5	0.475			0.670	0.670	0.670	0.670
*Correlations*							
Hyperactivity							
OE	0.327						
PE	0.265	*− .040*					
ED at 12	0.219	0.253	*− 0.004*				
ED at 15	*0.043*	0.208	*0.011*	0.308			
ED at 17	0.095	0.214	*0.046*	0.315	0.698		
ED at 20	*− .015*	0.142	*0.023*	0.272	0.598	0.759	
ω	0.801	0.619	0.686	0.680	0.754	0.754	0.754

^*1*^ Even though the unstandardized factors loadings are invariant over time, the standardized factor loading of the first ED item is different at Time 1 due to the lack of invariance of its uniqueness; OE = overeating; PE = picky eating; ED = eating disorders; ω = composite reliability coefficient (McDonald, 1970); Non statistically significant (p ≤ .05) parameters are in italics

## Latent curve modeling (LCM)

**Fig. 1:**
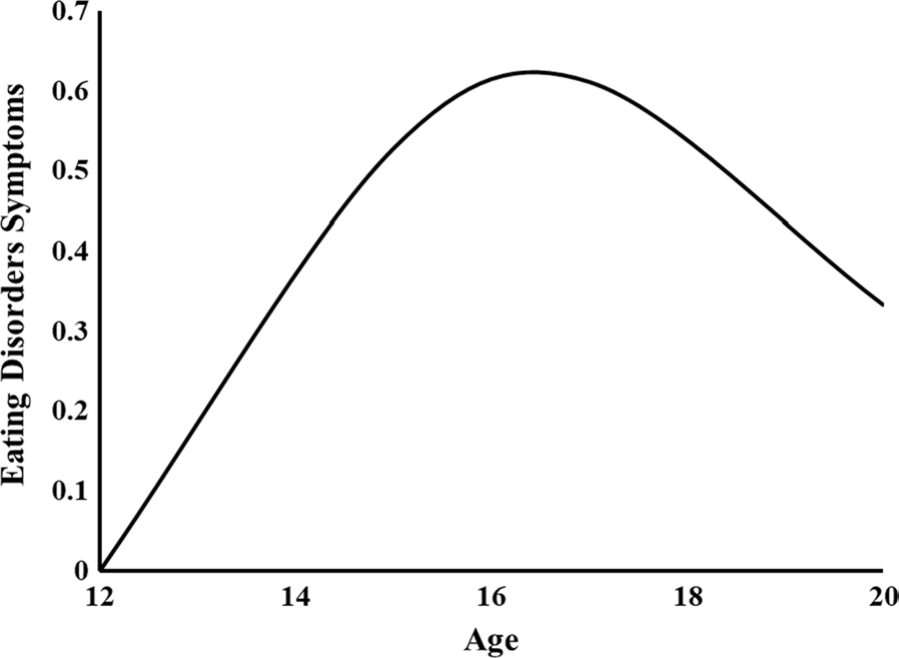
Estimated Quadratic Trajectories of Eating Disorders Symptoms. Y axis represents the estimated average levels of eating disorders symptoms, starting from a sample mean set to 0 at age 12 for identification purposes

The fit of the two alternative LCM estimated for the repeated measures of ED symptoms are reported in the middle section of Table 1. Whereas the fit of the linear solution failed to achieve acceptability standards according to the TLI fit index, that of the quadratic model was excellent according to the TLI and RMSEA fit indices and acceptable according to the TLI, consistent with the presence of curvilinear trajectories. An examination of the parameter estimates of the quadratic solution was consistent with this interpretation, revealing statistically significant linear (*M* = 0.257; *SE* = 0.034; *p* ≤ 0.01) and quadratic (*M* = − 0.027; *SE* = 0.004; *p* ≤ 0.01) slope factors. The shape of the ED trajectories estimated as part of this quadratic model, which was retained for further stages of analyses, is illustrated in Fig. 1. These results are consistent with the presence of a sharp increase in ED symptoms between the ages of 12 and 15, followed by a flattening out of this increase and a slight decrease until the age of 17, and then by a decrease until the age of 20. These results are consistent with the latent means estimated as part of our preliminary measurement models, while showing an inflexion point located around 16 years.

## Predictive analyses

**Fig. 2:**
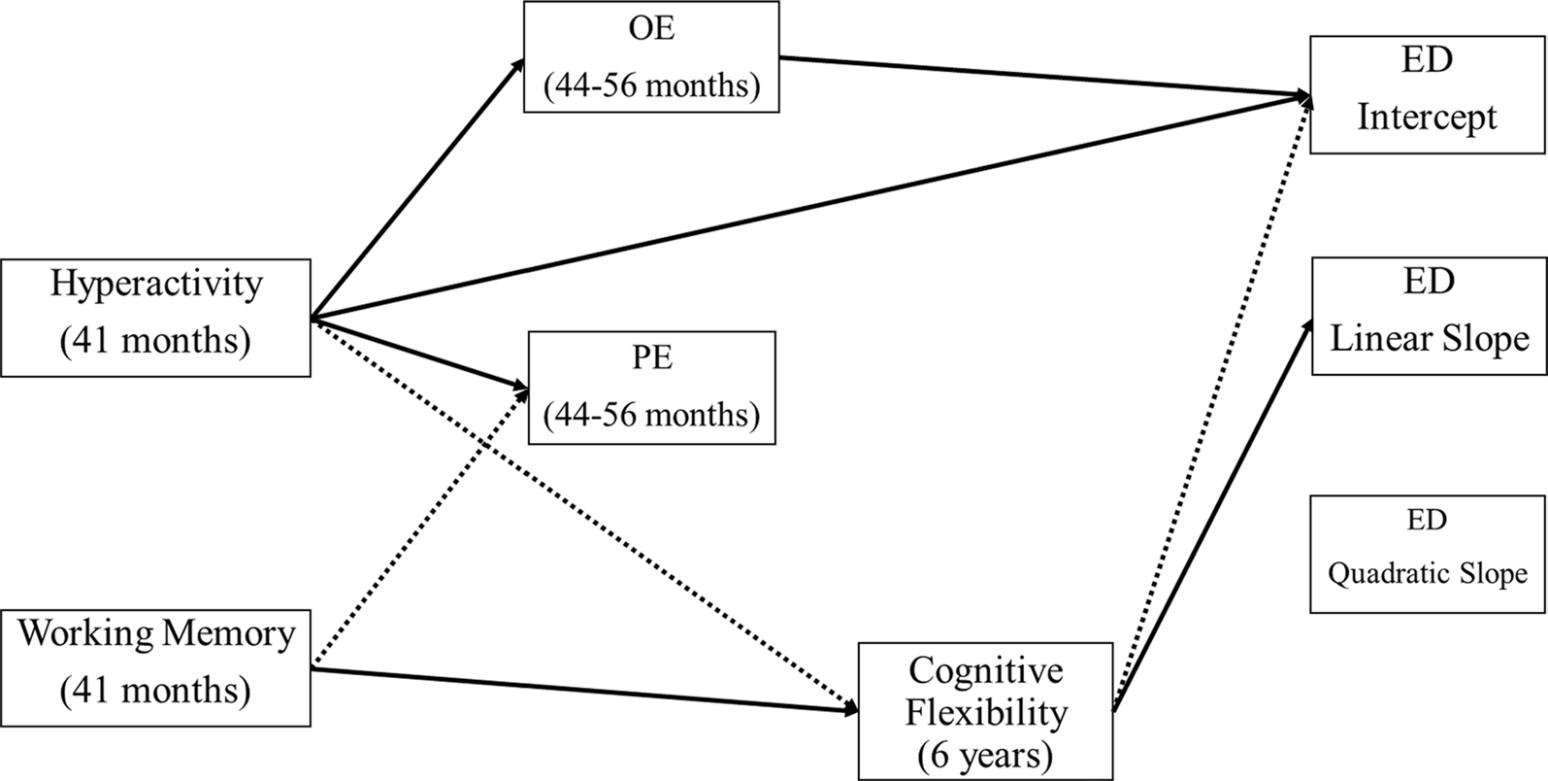
Graphical Representation of the Statistically Significant Direct Paths Note. full Arrows = positive associations; dotted Arrows = negative associations

The fit of the alternative predictive models is reported in the bottom of Table 1 and reveals an excellent level of fit for both models. These results show that the fit of the full mediation model is virtually identical to that of the partial mediation model. However, parameter estimates indicate a statistically significant direct association between hyperactivity and the intercept factor of the ED trajectories, leading us to retain the model of partial mediation. The results from this model of partial mediation are reported in Table 3. These results show that hyperactivity and overeating were both positively associated with the intercept of the ED symptoms trajectory. Cognitive flexibility was negatively associated with the intercept and positively associated with the linear slope factor. None of the predictors were significantly related to the quadratic slope factor. Although working memory and picky eating were not significantly associated with any of the growth factors, working memory was negatively associated with picky eating and positively associated with cognitive flexibility. Lastly, hyperactivity was positively associated with overeating and picky eating. These results are graphically presented in Fig. 2.

**Table 3:** Predictive Results

Predictors	*b*	*SE*	β
*Direct effects on the intercept factor*			
Hyperactivity	0.077	0.034*	0.255
Overeating	0.194	0.066**	0.412
Picky Eating	− 0.037	0.043	− 0.092
Working Memory	0.009	0.031	0.022
Cognitive Flexibility	− .0073	0.022**	− .0319
*Direct effects on the linear slope factor*			
Hyperactivity	− 0.030	0.016	− 0.200
Overeating	0.015	0.032	0.063
Picky Eating	0.026	0.022	0.128
Working Memory	− 0.013	0.016	− 0.070
Cognitive Flexibility	0.024	0.011*	0.213
*Direct effects on the quadratic slope factor*			
Hyperactivity	0.002	0.002	0.109
Overeating	− 0.002	0.004	− 0.069
Picky Eating	− 0.002	0.003	− 0.082
Working Memory	0.002	0.002	0.076
Cognitive Flexibility	− 0.002	0.001	− 0.138
*Direct effects on overeating*			
Hyperactivity	0.208	0.026**	0.324
Working Memory	− 0.042	0.029	− 0.050
*Direct effects on picky eating*			
Hyperactivity	0.195	0.027**	0.262
Working Memory	− 0.056	0.041*	− 0.059
*Direct effects on cognitive flexibility*			
Hyperactivity	– 0.171	0.043**	− 0.130
Working Memory	0.171	0.051**	0.101

* *p* ≤ .05; ** *p* ≤ .01; b = unstandardized regression coefficient; SE = standard error of the coefficient; β = standardized regression coefficient

These results suggest the possible presence of only three of the expected indirect associations: (a) a positive indirect association between hyperactivity – > overeating – > initial levels of ED symptoms; (b) a positive indirect association between hyperactivity – > cognitive flexibility – > initial levels of ED symptoms; (c) a negative indirect association between hyperactivity – > cognitive flexibility – > linear slope of ED symptoms. They also suggest two unexpected indirect associations: (a) a negative indirect association between working memory – > cognitive flexibility – > initial levels of ED symptoms; (b) a positive indirect association between working memory – > cognitive flexibility – > linear slope of ED symptoms. All indirect paths were statistically significant (Table 4).

**Table 4:** Statistically significant indirect effects from hyperactivity to ED symptoms growth factors

Pathway	Indirect effect	Bootstrap CI
Hyperactivity→Overeating→ED Intercept	0.040	0.016;0.080
Hyperactivity→Cognitive flexibility→ED Intercept	0.012	0.005; 0.026
Hyperactivity→Cognitive flexibility→ED Linear Slope	− 0.004	− 0.011; − 0.001
Working memory →Cognitive flexibility→ED Intercept	− 0.013	− 0.029; − 0.005
Working memory →Cognitive flexibility→ED Linear Slope	0.004	0.001; 0.011

Bootstrap CI = bias-corrected bootstrapped confidence intervals

## Discussion

The main objective of this longitudinal study was to examine the contribution of early childhood hyperactivity, eating behaviours, and executive functions to the development and course of ED symptoms from early adolescence to young adulthood. Our results indicated that ED symptoms tended to follow a quadratic (curvilinear) trajectory over the course of adolescence, characterized by a marked increase in ED symptoms between 12 and 15 years, followed by a decrease until 20 years old. The shape of these trajectories aligns with results obtained in previous studies on the evolution of ED symptoms [5, 44, 48]. The decrease in ED symptoms observed at the end of adolescence could possibly be due to changes in the relative prevalence of various types of ED symptoms, as binge-eating symptoms tend to become more common with age (2). Moreover, this decrease suggests that at least a subset of youth with ED symptoms, possibly those presenting subclinical symptoms, may progressively learn to better control these symptoms as they get older.

The present study complemented previous research on the early childhood precursors of ED by focusing on the role of hyperactivity, eating behaviours, and executive functions. Our results showed that higher levels of hyperactivity, lower levels of cognitive flexibility, and higher levels of overeating behaviours in childhood tended to predict higher initial levels of ED symptoms in early adolescence (12 years). Additionally, higher levels of cognitive flexibility were also associated with a higher rate of increase in ED symptoms trajectories during adolescence. At least part of this unexpected result may reflect the multivariate nature of our analyses, and in particular the correlation (*r* = 0.485) observed between the initial levels and the linear slope of ED symptoms trajectories. More specifically, this result needs to be interpreted considering the negative associations between cognitive flexibility and the initial levels of ED symptoms. Given that youth with low levels of cognitive flexibility already tend to start adolescence with higher levels of ED symptoms, there might be less room for their symptoms to increase over time. These findings could be related to cognitive flexibility being associated differently to certain EDs, as restrictive ED presentations such AN tend to emerge earlier than recurrent binge-eating ED presentations such as binge eating disorder [49, 50]. Finally, the unexpected result could be reflective of the different facets of cognitive flexibility being conflated into one measure of the construct, as these have been found to relate differently to EDs [51]. Given that the previously reported associations between low cognitive flexibility and the clinical severity of AN have been generally limited to clinical populations [20–22], the present results are important in suggesting that the preceding associations might be more complex among non-clinical populations of adolescents. Considering the mixed results associating cognitive flexibility and ED symptoms, more research will be required to better unpack the associations before making conclusions about using this outcome in early detection.

In relation to hyperactivity, most of the previous research on the associations between ADHD and EDs such as BN and BED has focused on impulsivity and its cross-sectional association with binge-eating or purge behaviours [52]. Our results complement the preceding findings by showing that early childhood hyperactivity, a behavioural facet of ADHD, does also play a role in the emergence of higher levels of predicts ED symptoms (including both restrictive and binge eating or purging symptoms) in early adolescence.

Both overeating and cognitive flexibility in childhood were found to partially mediate the association between early childhood hyperactivity and initial level of ED symptoms in early adolescence. Cognitive flexibility also mediated the association between early childhood hyperactivity and increase in ED symptoms across adolescence. In contrast, picky eating, although related to hyperactivity, did not mediate these associations, and seemed to share no associations with ED symptoms. Globally, these results support those from previous studies reporting positive cross-sectional and longitudinal associations between hyperactivity and overeating [14, 15, 17–19]. Our results thus suggest that early hyperactivity may lead children to overeat in childhood, possibly because of their lack of impulse control, which then places them at an increased risk of experiencing high levels of ED symptoms in adolescence. Overeating in childhood and its association with obesity may be especially linked to future EDs through the development of body image concerns [53, 54]. In relation to cognitive flexibility, our results also generally support the previously reported presence of cognitive impairments among people with ADHD [24–26]. However, our results add to the previous body of knowledge by suggesting that cognitive flexibility may be more than a simple correlate of ED symptoms and may rather represent an antecedent of their development. Interestingly, our results uncovered an indirect effect whereby working memory indirectly contributes to the development of ED symptoms through its documented positive associations with cognitive flexibility [55]. As key components of executive functioning, impairments of working memory and cognitive flexibility together have been linked to emotion regulation and self-regulatory mechanisms [56, 57]. It is likely that a certain cognitive profile, rather than isolated cognitive functions, could lead to increased risk of ED symptoms.

Additional results replicated past differences in prevalence of ED symptoms [2, 4, 5] and hyperactivity [42, 43], and supported the equivalence of the identified developmental mechanisms across boys and girls (Additional file 2). High and more pronounced quadratic trajectories for girls appear to indicate more rapid development of symptoms in early adolescence, which could be due to the stronger influence of puberty on ED risk and earlier pubertal age than in boys [58, 59]. This suggests that early detection and intervention efforts guided by the results are likely to generalize to samples of at-risk boys and girls.

## Strengths and limitations

Strengths of the study are that the study was conducted in a well-documented cohort sample followed prospectively from birth to adulthood. Additionally, the repeated assessments of ED symptoms from adolescence to adulthood made it possible to not only predict symptom severity, but also the evolution of ED symptoms over time. Furthermore, our reliance on fully latent models means that all associations uncovered in the present study can be considered to be controlled for unreliability. Still, some limitations also need to be considered. First, as is always the case in longitudinal cohort studies, missing responses and missing times points were present, and relatively high for the adolescent timepoints. In this regard, even though it was necessary to rely on WLSMV estimation to handle the binary and ordinal nature of our indicators, this estimator relies on a slightly less efficient way of handling missing responses than full information algorithms implemented with maximum likelihood estimation [40, 41]. However, both types of algorithms have a similar rate of efficacy and are more robust to the effects of missing responses than most available alternatives [40, 41]. Furthermore, the measure used to assess ED symptoms (SCOFF) is a self-report questionnaire that only assesses a few symptoms through a binary rating scale, which may have resulted in a loss of variability and precision. The lack of comprehensive specific measures for AN or avoidant/restrictive food intake disorder (ARFID), which have both been associated with picky eating in previous research [13, 60], may also explain the lack of findings relating picky eating to ED symptoms. Additionally, low scale-score reliability of the working memory measure may be of concern. However, it is not uncommon for neuropsychological tests with few trials to have low reliability estimates [61]. Finally, the design of our study and the nature of the variables contribute to our inability to completely differentiate between the cognitive impairments as risk factors for EDs, or as early signs of the disorder.

## Future directions

The current study was based on a community sample. Future studies in patient populations are needed to study the clinical relevance of our findings. Due to the counter-intuitive finding regarding cognitive flexibility, there is a need for replication of the current findings to better disentangle associations between early cognitive flexibility and EDs. Furthermore, in terms of cognitive measures, the current study only focused on cognitive flexibility and working memory. Future research should study whether results can be generalized to other cognitive domains such as attention and inhibitory control. Designing and testing interventions aimed to improve relevant cognitive domains and examining possible impacts on risk for ED should be tested in future studies.

## Conclusion

Using a prospectively longitudinal design, including measures from early childhood to young adulthood, our study is the first to identify childhood hyperactivity, overeating and cognitive flexibility as possible precursors of the onset of ED symptoms in adolescence. Providing future replication of the findings, the work could inform preventive intervention programs for EDs. This could potentially mean targeting children who present certain risk behaviours (i.e., low working memory, low cognitive flexibility, high hyperactivity, high overeating), and starting these programs before the age of 12, as ED symptoms seem to increase afterwards.

### Supplementary Information

**Additional file1**: Preliminary measurement models and longitudinal measurement invariance. This document provides detailed statistical information on the estimation of preliminary measurement models and their psychometric properties. It also includes the specific sequence of estimation used to assess longitudinal invariance of ED symptoms development over the four timepoints and its results (i.e., model fit indices, change in model fit).

**Additional file2 **: Assessing possible sex differences. This document provides detailed information on additional statistical analyses conducted to assess possible sex differences in measurement invariance, in the estimation of latent curve models, and equivalence of the predictions reported in the main manuscript. This includes a detailed table with model fit indices and model fit change of each measurement models, and a figure demonstrating the estimated latent curve models by sex.

## Data Availability

Available upon request.

## Abbreviations

ED: Eating disorders
AN: Anorexia nervosa
ADHD: Attention Deficit Hyperactivity Disorder
BN: Bulimia nervosa
EDNOS: Eating Disorder Not Otherwise Specified
QLSCD: Quebec Longitudinal Study of Child Development
SCOFF: Sick, Control, One stone, Fat, Food
WLSMV: Weighted least square estimation
LCM: Latent curve model
TLI: Tucker-Lewis index
RMSEA: Root mean square error of approximation
ARFID: Avoidant/Restrictive Food Intake Disorder

## Footnotes

1 For exploratory purposes, we tested an alternative model in which cognitive flexibility was positioned as a mediator of the relations between overeating and picky eating and the growth factors. We found no evidence that this was the case.
