# Supplementary material for: Childhood hyperactivity, eating behaviours, and executive functions: Their association with the development of eating-disorder symptoms in adolescence
Source: J Eat Disord. 2023 Oct 13;11:183. doi: 10.1186/s40337-023-00902-z (PMC10571422; doi:10.1186/s40337-023-00902-z)
Supplement: Supplementary file 1 — Additional file1: Preliminary measurement models and longitudinal measurement invariance. This document provides detailed statistical information on the estimation of preliminary measurement models and their psychometric properties. It also includes the specific sequence of estimation used to assess longitudinal invariance of ED symptoms development over the four timepoints and its results (i.e., model fit indices, change in model fit). [file 40337_2023_902_MOESM1_ESM.pdf]

## Preliminary Measurement Models & Longitudinal Measurement Invariance

We estimated preliminary measurement models to verify the psychometric properties of our measures. More specifically, we first estimated a seven-factor confirmatory factor analytic (CFA) model in which ratings of hyperactivity (five items), overeating (two items) and picky eating (two items) were each represented by one factor defined by their a priori indicators, and time-specific ratings of ED (five items) was represented by a series of five factors (one per time point) defined by their a priori indicators. All factors were allowed to correlate, and *a priori* correlated uniquenesses were included between the matching indicators of ED utilized at the different time points to avoid inflated stability estimates [1]. Because the overeating and picky eating factors were each defined by only two indicators, essentially tau-equivalent constraints were imposed on their factor loadings (constraining them to equality) to achieve local identification [2, 3].

We examined the longitudinal measurement invariance of the ED ratings in the following the sequence, recommended for binary items [4]: (1) configural (same model with no additional constraint, corresponding to our global measurement model); (2) strong (equal factor loadings and response thresholds over time; test of the invariance of the loadings and thresholds cannot be separated for binary items); (3) strict invariance (equal item uniquenesses); (4) latent variances invariance (equal factor variances); (5) latent mean invariance (equal factor means). Model fit and measurement invariance were assessed using the comparative fit index (CFI), the Tucker-Lewis index (TLI), and the root mean square error of approximation (RMSEA) and its 90% confidence interval. Adequate model fit was indicated by CFI and TLI values > .90 and RMSEA values < .08, while excellent fit was indicated by CFI and TLI values > .95 and RMSEA values < .06 [5, 6]. A decrease of CFI and TLI > .01 and an increase of RMSEA **> .**015 relative to the

previous model in the sequence were used as evidence of measurement invariance [7, 8]. Chi-square difference tests for WLSMV estimation were calculated using the Mplus DIFFTEST function [4]. However, chi-square and chi-square differences tests are not interpreted given their oversensitivity to sample size and minor misspecifications [5, 6]. The most invariant model (up to strict to allow for the unconstrained estimation of the growth trajectories) was used as input for our main analyses. However, the model of strict invariance was not supported by the data ($\Delta$CFI = -.044, $\Delta$TLI = -.049). Examination of the modification indices associated with the model of strict invariance and of the parameter estimates from the previous model of strong invariance suggested that this non-invariance was limited to the uniqueness of the first ED item (i.e., purging), which was slightly less reliable at Time 1 (12 years, uniqueness = .892) than at the latter time points (15, 17, and 20 years, uniqueness = .528). After relaxing the equality constraints on this uniqueness, the resulting model of partial strict invariance was supported by the data. The final model of latent mean invariance was also not supported by the data, and parameter estimates from the previous model indicated that ED levels underwent a drastic increase between 12 ($M$ = 0 in standardized units) and 15 ($M$ = .727 SD units higher than at 12 years), kept on increasing slightly until 17 ($M$ = .765 SD units higher than at 12 years), before starting to decrease until 20 ($M$ = .605 SD units higher than at 12 years), consistent with a quadratic (curvilinear) trajectories.

References


1. Brooks BL, Sherman EMS, Iverson GL, et al (2011) Psychometric foundations for the interpretation of neuropsychological test results. In: The little black book of neuropsychology: A syndrome-based approach. Springer Science + Business Media, New York, NY, US, pp 893–922

2. Little TD, Cunningham WA, Shahar G, Widaman KF (2002) To Parcel or Not to Parcel: Exploring the Question, Weighing the Merits. Struct Equ Model Multidiscip J 9:151–173. https://doi.org/10.1207/S15328007SEM0902_1

3. Little TD, Lindenberger U, Nesselroade JR (1999) On selecting indicators for multivariate measurement and modeling with latent variables: When "good" indicators are bad and "bad" indicators are good. Psychol Methods 4:192–211. https://doi.org/10.1037/1082-989X.4.2.192

4. Mplus User Guide

5. Cutoff criteria for fit indexes in covariance structure analysis: Conventional criteria versus new alternatives. https://www.tandfonline.com/doi/epdf/10.1080/10705519909540118?needAccess=true&role=button. Accessed 8 Dec 2022

6. Marsh HW, Hau K-T, Grayson D (2005) Goodness of Fit in Structural Equation Models. In: Contemporary psychometrics: A festschrift for Roderick P. McDonald. Lawrence Erlbaum Associates Publishers, Mahwah, NJ, US, pp 275–340

7. Chen FF (2007) Sensitivity of goodness of fit indexes to lack of measurement invariance. Struct Equ Model 14:464–504. https://doi.org/10.1080/10705510701301834

8. Cheung GW, Rensvold RB (2002) Evaluating goodness-of-fit indexes for testing measurement invariance. Struct Equ Model 9:233–255. https://doi.org/10.1207/S15328007SEM0902_5
