# Supplementary material for: Childhood hyperactivity, eating behaviours, and executive functions: Their association with the development of eating-disorder symptoms in adolescence
Source: J Eat Disord. 2023 Oct 13;11:183. doi: 10.1186/s40337-023-00902-z (PMC10571422; doi:10.1186/s40337-023-00902-z)
Supplement: Supplementary file 2 — Additional file2 : Assessing possible sex differences. This document provides detailed information on additional statistical analyses conducted to assess possible sex differences in measurement invariance, in the estimation of latent curve models, and equivalence of the predictions reported in the main manuscript. This includes a detailed table with model fit indices and model fit change of each measurement models, and a figure demonstrating the estimated latent curve models by sex. [file 40337_2023_902_MOESM2_ESM.pdf]

Assessing possible sex differences

Supplementary sex differences results are reported in Supplementary Table 1. In terms of measurement invariance, these results replicated those from the main analyses in supporting the invariance of most model parameters. As in our main analyses, equivalence of residual variances (strict invariance) was not entirely supported, but a model of partial strict invariance was supported for most item uniquenesses (ED item 1 was still less reliable at Time 1 – 12 years – than at later time points in both groups). This means that residual variances of most ED items were equivalent across boys and girls, with a subset of ED items proved to have a level of reliability that slightly differed across boys and girls. As for our main analyses, the invariance of the latent means was not supported, consistent with the presence of quadratic trajectories that were characterized by a slightly higher level and rate of increase among girls relative to boys, and with the presence of higher levels of hyperactivity among boys. The time-specific residuals and latent variance-covariance of the latent curve model were also equivalent across sex, whereas the means of the trajectories differed, indicating higher and more pronounced quadratic trajectories for girls, as illustrated in the Supplementary Figure 1. Finally, all predictions were equivalent for boys and girls, supporting the generalizability of our main conclusions.

Supplementary Table 1

Results from the Sex-Related Comparisons

Model	c^2 (df)	CFI	TLI	RMSEA	90% CI	CM	DCFI	DTLI	DRMSEA	Dc^2 (df)
Measurement invariance across sex and time										
1. Configural	803.520 (652)*	.977	.971	.015	.011; .019	---	---	---	---	---
2. Weak invariance (non-binary items)	813.189 (658)*	.977	.971	.015	.012; .019	1	.000	.000	.000	10.401 (6)
3. Essential tau-equivalence (overeating and picky eating)	820.137 (660)*	.976	.970	.016	.012; .019	2	-.001	-.001	+.001	6.555 (2)
4. Strong invariance (all items)	896.163 (691)*	.969	.964	.017	.014; .020	3	-.007	-.006	+.001	82.302 (31)*
5. Strict invariance	1387.944 (735)*	.901	.891	.030	.027; .032	4	-.068	-.073	+.013	545.003 (44)*
5a. Partial strict invariance	971.447 (728)*	.963	.959	.018	.015; .021	4	-.006	-.005	+.001	87.640 (37)*
6. Longitudinal correlated uniquenesses invariance	1043.221 (758)*	.957	.954	.019	.016; .022	5a	-.006	-.005	+.001	101.019 (30)*
7. Latent variance-covariance invariance	1078.036 (774)*	.954	.952	.020	.017; .023	6	-.003	-.002	+.001	34.850 (16)*
8. Latent means invariance	1734.392 (784)*	.856	.851	.035	.033; .037	7	-.098	-.101	+.015	540.992 (10)*
Equivalence of the latent curve models across sex										
L1. Baseline quadratic model (all free)	567.571 (349)*	.928	.921	.028	.023; .032	---	---	---	---	---
L2. Equivalent time-specific residuals	590.182 (353)*	.921	.915	.029	.024; .033	L1	-.007	-.006	+.001	23.958 (4)*
L3. Equivalent growth factors variances and covariances	584.093 (359)*	.925	.921	.028	.023; .032	L2	+.004	+.006	-.001	7.311 (6)*
L4. Equivalent growth factor means	880.541 (362)*	.828	.820	.042	.038; .045	L3	-.097	-.101	+.014	247.357 (3)*
Equivalence of the predictions across sex										
P1. Baseline partial mediation (all free)	1217.996 (868)*	.948	.944	.020	.017; .023	---	---	---	---	---
P2. Equal predictions	1218.504 (889)*	.951	.948	.019	.017; .022	P1	+.003	+.004	-.001	19.083 (21)*

Note. * $p \leq .01$; c^2 = chi-square test of exact fit; df = degrees of freedom; CFI = comparative fit index; TLI = Tucker-Lewis index; RMSEA = root mean square error of approximation; 90% CI: 90% confidence interval for the RMSEA; CM = comparison model; D = change in model fit relative to the CM.

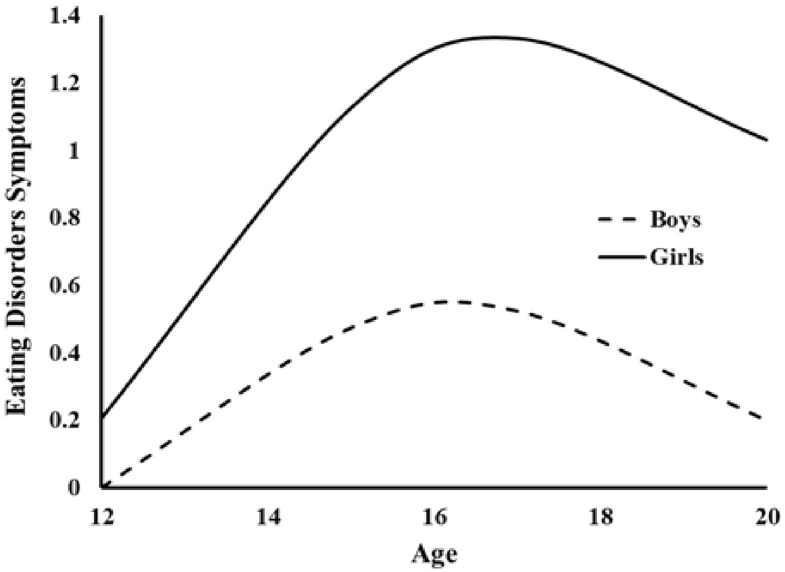

Supplementary Figure 1. *Boys and Girls Estimated Quadratic Trajectories of Eating Disorders Symptoms .*

Note. Y axis represents the estimated average levels of eating disorders symptoms, starting from a sample mean set to 0 at age 12 for identification purposes.
